# Being (a) patient—navigating desire through online communities in assisted reproductive technology

**DOI:** 10.3389/fsoc.2025.1683860

**Published:** 2026-01-08

**Authors:** Alessandra Decataldo, Elena Andreoni

**Affiliations:** Department of Sociology and Social Research, University of Milan-Bicocca, Milan, Italy

**Keywords:** assisted reproductive technology (ART), online communities, netnography, parental desire, social stigma

## Abstract

**Introduction:**

Italy faces a significant demographic decline, with Assisted Reproductive Technology (ART) becoming a critical response to infertility. Despite its medicalization, ART remains socially ambivalent, marked by stigma and emotional isolation. Online platforms provide spaces for navigating this complex experience. This study, part of the PRIN PNRR Fertility Over fortIES (FORTIES) project, explores how digital communities dedicated to ART serve as socio-technical environments for articulating and transforming reproductive desire, focusing on stigma, knowledge sharing, and emotional support.

**Materials and methods:**

We conducted a netnographic study across three digital spaces: a public ForumFree forum, Facebook groups, and a Telegram chat. Data were collected via web scraping, manual archiving, and platform-specific data extraction tools. The analysis employed a grounded theory approach in Nvivo software, focusing on themes of medical knowledge, emotional support, and the performativity of parenthood.

**Results:**

The netnographic analysis revealed discursive practices and relational strategies that shape the experience of ART pathways in online communities. A central theme was the female predominance on the platforms, driven by women’s higher participation in seeking health information and the uneven medical burden in ART. Digital communities legitimize experiences by offering emotional support and validation, normalizing vulnerability and anxiety common in ART journeys. The sharing of clinical and personal experiences creates a hybrid form of situated knowledge, bridging technical language with lived experience. Parenthood is presented as a symbolic, uncertain process, anticipated before pregnancy. Platforms foster ritualized waiting practices, helping manage uncertainty. The analysis of affordances showed how platform features shape specific interaction dynamics.

**Discussion:**

ART is a complex, multimodal experience involving clinical, emotional, and relational dimensions. In Italy, infertility and medicalized parenthood are often stigmatized, pushing these experiences into digital spaces where platforms such as Telegram, Facebook, and ForumFree provide crucial support and community. These platforms allow users to share personal and medical experiences, manage uncertainty, and create a sense of belonging. However, challenges such as access barriers, misinformation, and the reinforcement of normative expectations remain. The study highlights that ART is not just a medical process but is deeply shaped by digital storytelling and collective participation, influencing how reproductive subjectivities are constructed and understood.

## 1 Introduction

Italy is among the countries most affected by demographic decline, with one of the lowest birth rates in Europe. According to Istat—Istituto Nazionale di Statistica (2025), Italy’s total fertility rate in 2024 dropped to 1.18 children per woman, marking a continued decline from the previous decade. Eurostat (2025) confirms that Italy ranks among the lowest in the European Union in terms of fertility, and the average age at first childbirth reached 32.6 years in 2024. This phenomenon of postponed parenthood is the result of a complex interplay of structural, economic, and cultural factors, including job insecurity, gender inequalities in caregiving, housing precarity, and a persistent lack of substantial welfare policies supporting family formation.

Such dynamics reflect broader trends identified in demographic and sociological literature, which describe fertility postponement as shaped by intersecting biosocial clocks and neoliberal precarity (Billari et al., 2011; Wilkinson and Rouse, 2022; Yopo Díaz, 2021a,b).

Against this backdrop, Assisted Reproductive Technologies (ART) have emerged not only as medical responses to biological infertility but also as socio-technical solutions to the broader temporal disjunction between reproductive desire and feasibility.

According to the most recent CeDAP—Certificato di assistenza al parto (2023) report by the Italian Ministry of Health, 3.9% of all live births in Italy are the result of ART, with higher concentrations in urban centers, among women with medium-high educational attainment, and among women aged over 35 (it is 19.2% for mothers over the age of 40).

ART, therefore, does not operate in a vacuum: it is embedded in a broader reconfiguration of reproductive temporality, where biopolitical and biomedical infrastructures reshape intimate life courses. Thus far, despite its diffusion and normalization, ART remains a socially and symbolically ambivalent phenomenon in Italy. Stigma, silence, and emotional isolation still mark the experience of infertility and assisted reproduction, rendering it at once hyper-visible in political discourse (especially in relation to bioethics and legislation) and under-articulated in everyday social interactions. As De Sanctis et al. (2020) argue in their sociological reading of motherhood, ART challenges normative timelines and calls into question cultural definitions of reproductive success, often without corresponding shifts in institutional or everyday discourses.

The lived experience of ART is profoundly medicalized. Clinical procedures, hormone treatments, cycles of ovulation monitoring, and embryo transfers structure the lives of those undergoing assisted reproduction. Yet ART is also a deeply affective and intimate experience, characterized by vulnerability, hope, disappointment, and persistence. This juxtaposition creates a paradox: while ART subjects are immersed in a highly technologized and protocolized environment, they often lack spaces to articulate the emotional and social dimensions of their journey (Kashyap and Tripathi, 2025).

The rise of digital platforms has profoundly transformed how intimate, health-related, and socially sensitive experiences are shared and discussed. This is particularly evident in the context of ART, which unfolds in a multimodal manner (Garcia et al., 2009). Offline, patients navigate clinical environments, interact with physicians and embryologists, and manage their embodied responses to treatment. Online, they seek out spaces where they can narrate, question, compare, and share their experiences. These digital arenas become extensions and amplifiers of the ART journey, offering opportunities to navigate ambiguity, construct meaning, and connect with others facing similar challenges. The multimodal nature of ART, therefore, reflects its ontological complexity as both a biomedical intervention and a socio-cultural process. As noted by Santoro (2024), the articulation of maternal identity in the era of social media cannot be separated from the ways in which women navigate the visibility, aesthetics, and temporality of digital platforms, often oscillating between exhibition and discretion. Similarly, Wilkinson et al. (2023) describe how participants undergoing ART experienced alienation in institutional settings but found alternative forms of support, recognition, and negotiation in relational spaces—highlighting the fluid interplay between offline and digital coping mechanisms.

Digital platforms have increasingly emerged as crucial sites for navigating ART. They do not simply supplement clinical information but constitute discursive and affective infrastructures that mediate users’ engagement with their reproductive trajectories. Digital spaces such as ForumFrees, social media platforms like Facebook with its groups, and messaging apps such as Telegram are not neutral containers of content, but rather arenas in which community norms, epistemologies, and relational configurations are continuously produced and reproduced.

These online spaces fulfill multiple and intersecting functions. First, they offer validation and legitimation for experiences often marginalized or misunderstood in offline settings. Second, they serve as knowledge repositories, enabling the exchange of information about medical procedures, pharmacological treatments, clinics, and bodily sensations. Third, they foster the discursive construction of alternative models of parenthood, including single motherhood by choice or gamete donation, challenging dominant familial and geneticist narratives. Fourth, they ritualize key moments in the ART process, most notably the “two-week wait” between embryo transfer and pregnancy testing, transforming passive temporality into an active, collective practice. Individuals construct reproductive meaning not only through clinical outcomes but through “reproductive careers” (Johnson et al., 2018, 2023)—ongoing narratives shaped by class, age, gender, and temporality.

Each digital environment contributes differently to these functions. For example, ForumFrees, with their archival logic and thematic threads, provide structured spaces for consultation and narrative accumulation. Social media platforms like Facebook balance real-time interaction with post permanence, allowing for both affective exchange and information retrieval. Messaging apps such as Telegram chats, with their immediacy and intimacy, facilitate informal, emotional, and dynamic conversation. These dynamics resonate with what Segatto et al. (2023) describe as the creation of “temporal bubbles” and support networks that give collective shape to highly individualized experiences of waiting, failure, and anticipation.

This study stems from the PRIN PNRR research project *FORTIES—Fertility over forties, after the age of 40*, a national initiative that investigates late fertility through a mixed-methods approach involving multiple research units. Within this broader framework, the study focuses on ART, exploring how parenthood is imagined and constructed along these reproductive trajectories. Special attention is given to the ways in which these experiences—often marginalized or rendered invisible in dominant public narratives—are collectively articulated, negotiated, and imbued with meaning within digital environments.

In particular, this study investigates how digital communities focused on ART function as socio-technical environments in which reproductive desire is expressed and transformed. The study presents a netnographic exploration of three digital spaces dedicated to ART: a public ForumFree forum, two Facebook groups, and a Telegram chat. The affordances influence not only communicative style, but also the degree of visibility, exposure, and participation possible in each environment.

This paper aims to explore: (1) the structural barriers—particularly linguistic and epistemic divides—that hinder effective communication in the context of ART and how these are reconfigured in digital interactions. (2) The role of digital technologies in shaping user interactions, epistemologies, and identity performances, and in reconfiguring power relations traditionally centered on medical authority. (3) The communication strategies and adaptive practices enacted by users to navigate clinical, emotional, and social uncertainties associated with ART.

This research contributes to a sociological understanding of ART as a multimodal, culturally coded, and digitally mediated phenomenon.

The study is structured into the following sections: the next section attempts to define the theoretical framework; the following one describes Materials and Methods, the third section presents the Results, and the final one outlines the Discussion and Conclusion.

## 2 Theoretical framework

Contemporary parenthood is marked by pluralism and contestation. Traditional norms centered on the heterosexual, biologically connected nuclear family now coexist with a plurality of parental arrangements, including single parenthood by choice, same-sex parenting, and kinship structures mediated by ART. These transformations reflect broader societal shifts toward the decentering of normative biogenetic ties and the emergence of parenthood as a negotiated, performative, and mediated identity (Thompson, 2005; Mamo, 2007). ART constitutes a paradigmatic locus of this shift, functioning not only as a biomedical intervention but as a socio-technical assemblage in which biological limits and social possibilities intersect, creating new configurations of legitimacy, kinship, and temporality.

Paradoxically, while family models are multiplying, the desire for parenthood remains rarely thematized, taken for granted, and scarcely discussed as a socio-cultural construct. The absence of a consciously thematized space for reflecting on the desire for motherhood/parenthood emerges as a constitutive trait of the ART ecosystem. Unlike, for instance, adoption—where the presence of the child’s interest requires explicit reflection on parenthood—in ART, the desire to become a parent remains an unquestioned axiom, reinforced by its overlap with reproductive vulnerability and clinical urgency. In this sense, online groups seem to act as echo chambers that reinforce the desire for parenthood and allow users to articulate and reframe their reproductive paths, proposing discussions that question the traditional model of filiation.

De Sanctis et al. (2020) emphasize that contemporary motherhood is shaped by intricate negotiations between embodied constraints, cultural scripts, and institutional logics. Motherhood becomes a temporally distributed and technologically mediated identity rather than a naturalized outcome of biological reproduction. As such, the maternal subject is fragmented across affective investments, clinical timelines, and socio-technical devices. Cossetta and Caliandro (2013) complement this view by showing how online narratives of motherhood—particularly those produced in blogs and ForumFrees—offer not merely autobiographical accounts, but cultural scripts that normalize certain ways of “being mother” while marginalizing others. These narratives are shaped by dominant tropes such as the “good mother,” the “warrior mother,” or the “mother by vocation,” and operate within algorithmic logics that privilege affectively intense and morally resonant content.

Building on this, Lee (2017) reveals how online infertility ForumFrees privilege narratives of perseverance and reproductive endurance, coalescing around the figure of the “persistent patient”—a subject who cycles through multiple rounds of ART, often despite emotional exhaustion and financial precarity. In these spaces, parental desire is constructed not as innate or fixed, but as dynamically shaped through affective economies of hope, failure, and resilience (Ahmed, 2004).

The entanglement of digital media and reproductive experiences calls for a reconsideration of human subjectivity in the age of “onlife,” as articulated by Floridi (2014), who sees human existence as increasingly entangled with digital technologies. This approach avoids dualism, rejecting a strict separation between the real and the virtual, and instead proposes a reading of interactions within digital groups as extensions, multiplications, and reconfigurations of contemporary reproductive and parenting practices. In Floridi’s conceptualization, the “onlife” condition implies that the boundaries between offline and online spheres have become so blurred that human beings must be understood as informational agents inhabiting an infosphere—an environment shaped and saturated by data, digital infrastructures, and mediated relations (Hallett et al., 2014).

Consequently, reproductive subjectivities and experiences—such as seeking support, exchanging medical advice, or narrating fertility journeys—occur within this hybridized space, where digital technologies are not merely tools but constitutive of the very conditions of being and relating. From this perspective, online communities dedicated to assisted reproduction are not simply virtual extensions of offline sociality but are part of the very fabric through which reproductive identities and practices are formed, negotiated, and transformed.

This interpretive framework aligns with recent scholarship in digital sociology, such as Lupton (2018) and Selwyn (2019), who explore how digital environments not only mediate but actively constitute subjectivities and socio-technical imaginaries. In particular, Lupton emphasizes the embodied and affective dimensions of digital interactions, highlighting how practices such as self-tracking, sharing personal health narratives, or engaging in peer-to-peer support foster forms of “digitally mediated embodiment.” These processes are central to how users experience and give meaning to health, illness, and care in the digital age. Similarly, Selwyn (2019) underscores the importance of examining the socio-material configurations through which digital technologies shape imaginaries of the self, the family, and the future—especially in contexts where technological mediation is intimately linked to questions of morality, identity, and authority. These contributions are essential for understanding how ART-related communities produce and stabilize meaning around kinship, gender roles, and legitimacy through iterative practices of sharing, documenting, and emotional validation. Within these spaces, the repeated circulation of narratives—such as *in vitro* fertilization (IVF) diaries, embryo transfer updates, or ultrasound images—does not merely reflect existing norms but also participates in the collective crafting of new templates of reproductive legitimacy and belonging. In doing so, these communities act as sites of both epistemic and emotional labor, where reproductive experiences are co-produced through digital affordances and the affective economies they sustain.

The boundaries between online and offline dissolve in ART practices, where digital infrastructures—from period tracking apps to support groups—synchronize with medical regimens and emotional rhythms. ForumFrees, social media platforms, and messaging apps become not merely communicative tools but spaces of affective labor and symbolic survival. Grothaus et al. (2023) document how public infertility videos on platforms like YouTube serve dual functions: they satisfy patient needs for information, empathy, and experience-sharing, while simultaneously de-tabooing infertility in broader society. Cossetta and Caliandro (2013) further argue that these online practices are not only expressive but normative: the very act of narrating becomes a way to legitimate one’s maternal identity in the face of biomedical and social uncertainty.

Digital communities thus operate as counterpublics that legitimize stigmatized experiences, often compensating for the silences and omissions of clinical encounters. These online assemblages provide “safe spaces” where grief, uncertainty, and shame are collectively re-signified through discursive practices of recognition, echoing Ahmed’s (2004) notion of affective economies. As participants share stories of failed cycles, pregnancy loss, or navigating insurance hurdles (Mayette et al., 2024), pain becomes socially intelligible and emotionally validated. Digital communities act as spaces of legitimation, offering participants the opportunity to share their vulnerability and feel seen and understood.

Knowledge circulation within ART communities is deeply situated. Drawing on Haraway’s (1988) epistemology of situated knowledge, users co-produce lay expertise that challenges biomedical authority. For Haraway, knowledge is always partial, embodied, and located—it emerges from specific positions and material conditions rather than from an alleged “view from nowhere.” Within ART-related online communities, this means that reproductive knowledge is generated through the intersection of personal experience, digital mediation, and social interaction, making each contribution a form of contextualized epistemic labor. Such knowledge practices resist the hierarchical divide between expert and layperson, instead fostering a pluralistic ecology of knowledges in which experiential accounts, emotional insights, and peer support attain epistemic legitimacy.

Clarke et al. (2003) refer to these collectively generated frameworks as “vernacular biologies”: interpretive schemas rooted in everyday experiences of the body, illness, and treatment. In the context of ART, vernacular biologies emerge through storytelling about hormone injections, embryo quality, or the emotional toll of treatment cycles. These narratives do not merely supplement biomedical discourse but often reframe it, providing alternative logics of causality, success, and care that reflect users’ embodied realities. Furthermore, the digital infrastructure of online communities amplifies the visibility and persistence of these knowledge forms, enabling iterative refinements, validation through collective affect, and circulation across platforms and networks. As such, these communities function not just as support systems but as epistemic collectives that actively participate in shaping the meanings, practices, and politics of reproductive technologies.

The cyber-ethnographic study by Lee (2017) shows how these lay knowledges are often constrained by implicit norms—e.g., the valorization of expensive and exhaustive ART cycles—which marginalize users who cannot conform to the “persistent patient” script. Beyond emotional support, users co-construct a complex body of experiential knowledge.

This includes interpretations of medical protocols, comparative evaluations of clinics, pharmacological regimens, and post-transfer symptoms. What emerges is a horizontal epistemology, in which experiential competence complements—and at times challenges—medical authority. This situated knowledge is generated through the relationship between users within a complex system of co-produced knowledge. Within these networks, emotions, advice, and experiential knowledge circulate—but so too does misinformation (Jiménez-Palomares et al., 2021; O’Connor and Madge, 2004). Cossetta and Caliandro (2013) explore how mothers narrate their experiences of maternity in online environments such as ForumFrees, blogs, and social media. Their netnographic study of over 13,000 online conversations reveals that these digital narratives serve not only as spaces of reassurance and community-building but also as vehicles for challenging dominant biomedical framings of pregnancy and motherhood, valorizing lay knowledge and resisting over-medicalization.

These discourses also contribute to a digital re-symbolization of kinship and temporality. Through storytelling, commenting, and collective rituals—such as “roll calls” or countdowns during the “two-week wait”—users perform not only parenthood, but new modes of belonging and legitimacy. The emotional cadence of ART, often framed as linear medical progression, is instead refracted into shared, affectively saturated temporalities. In this sense, waiting becomes an active, performative practice that reshapes chrononormative expectations around reproduction (Bach, 2022). A particularly relevant dimension concerns the temporality of ART, especially the collective and emotionally charged moment of waiting between the transfer and the pregnancy test. This intermediate time is collectively ritualized: users share daily symptom tracking, organize countdowns, and exchange messages of encouragement, transforming passive waiting into a shared and meaningful practice. Waiting becomes both an existential condition and a symbolic space for processing desire, fear, and hope.

In sum, digital identity within ART communities emerges as a profoundly performative and relational construct. Through affectively charged narrative tropes—what Caliandro and Gandini (2019) defines as “invisible hashtags”—users align themselves with shared trajectories of hope, loss, or achievement, contributing to the emotional intelligibility and collective rhythms of these spaces. However, such narrative structures not only enable connection and validation but also delineate the boundaries of visibility and belonging. As Lee (2017) points out, experiences that fall outside dominant discursive norms—such as discontinuing treatment, facing economic barriers, or choosing not to parent—often remain unspoken or are subtly marginalized. These exclusions highlight the normative underpinnings that continue to shape digital reproductive cultures, reminding us that online spaces, while seemingly inclusive, are also sites of boundary-making, affective regulation, and epistemic inequality.

To capture and analyze these digitally mediated rituals and emotional investments, netnography offers a critical lens for examining such socio-technical practices. It allows researchers to observe how users navigate these affective temporalities, co-construct meaning, and embed their reproductive experiences within broader digital cultures of care and anticipation. Netnography is here intended as “a new qualitative research methodology that applies an ethnographic approach to the study of cultures and online communities” (Kozinets, 2017, 374).

In the following pages, we present a study aimed at moving beyond an analysis of digital contexts as mere spaces for information gathering or emotional support by investigating the subjective motivations and social functions that drive users to actively participate in these communities. These are interpreted here as relational and discursive devices that enable the co-construction of reproductive desire, emotional agency, and alternative forms of parenthood.

As Kozinets (2017) posits, digital spaces are not mere reflections or extensions of offline life but autonomous fields of meaning-making, embedded with their own symbolic economies, interactional norms, and cultural grammars. This perspective is particularly crucial when examining online communities centered on ART, where users not only exchange information or seek comfort, but actively co-create shared narratives, emotional frameworks, and epistemic repertoires. These spaces are performative arenas in which reproductive subjectivities are not simply expressed but continuously negotiated and reconfigured.

Such a view necessitates adopting methodological principles such as “follow the medium” (Rogers, 2013) and “follow the natives” (Latour, 2005), which together foreground the relational and material dynamics of digital ethnography. Rogers urges researchers to attend closely to the specific affordances, architectures, and cultural logics of individual platforms: the persistent pseudonymity of long-standing fertility ForumFrees fosters intimate storytelling; the real-time flow of Telegram channels facilitates rapid emotional resonance and coordination; the semi-public structure of Facebook groups enables community curation, gatekeeping, and boundary work. These platform-specific conditions shape what is considered legitimate discourse, how support is articulated, and how temporalities of treatment, hope, and loss are collectively synchronized.

Conversely, Latour shifts the analytical gaze toward the vernacular epistemologies and situated practices of users themselves. Rather than applying fixed theoretical lenses onto the field, this principle invites researchers to explore how users categorize their experiences (e.g., “PUPO”—“pregnant until proven otherwise”), coin terms, create acronyms, and develop taxonomies of meaning that are deeply embedded in embodied experience and temporality. Within ART communities, these emergent categories often function as affective anchors and identity markers, enabling users to locate themselves within the complex landscape of reproductive trajectories. This emic approach is essential to understanding how knowledge, legitimacy, and emotional agency are distributed, contested, and validated within digital assemblages of reproduction.

## 3 Materials and methods

This study adopts a multi-sited, qualitative netnographic approach (Kozinets, 2010), informed by the epistemological principles of situated knowledge and digital subjectivity (Caliandro and Gandini, 2019).

Observing online reproductive communities allows us to investigate how medicalized experiences, parental desire, and intimate suffering are articulated across platform-specific affordances and discursive regimes.

Grounded in a qualitative approach, the research aims to explore in depth the collective meanings of the phenomenon, with the objective of capturing the representations attributed to the ART experience. The goal was to understand the co-construction of meaning of parenthood by individuals involved in ART pathways in Italy. The theoretical starting point was the research question: *What happens within online communities dedicated to ART and infertility?* with a particular focus on the discursive, narrative, and performative forms emerging around the dense theme of pregnancy and parenthood in digital contexts.

As previously mentioned, ART experiences are complex phenomena that intertwine clinical, symbolic, psychological, and ethical dimensions. In the Italian cultural context—marked by rigid norms, taboos, and stigma—these experiences are often pushed into invisibility. Within this scenario, digital spaces play a central role in compensation, expression, and collective reworking, acting as *liminal spaces* (Madge and O'Connor, 2005) where experiences that are not always shareable elsewhere can be voiced, or that require forms of exchange and response that cannot be fully addressed offline. Hence, the choice of netnography terms is aimed at exploring digital cultures through the observation of computer-mediated interactions. Netnography makes it possible to investigate how individuals construct, negotiate, and perform their identities and worldviews within structured and relational digital environments.

The specificity of digital ethnography lies in the setting of the online space. This particular setting presents distinct characteristics that must be considered when defining the research design, particularly with respect to methodological, technical, and ethical challenges.

### 3.1 Mapping and selection of online fields

The fieldwork began with a systematic mapping of Italian digital contexts dedicated to ART and infertility, conducted between February and March 2024 through an in-depth web exploration. The use of targeted keywords such as “Procreazione Medicalmente Assistita” (“Assisted Reproductive Technology”), “PMA” (“ART”), “fertilità” (“fertility”), “infertilità” (“infertility”), “fecondazione assistita” (“assisted fertilization”), “IVF,” “ICSI,” “mamma/mamme” (“mum/mums”), and “gravidanza” (“pregnancy”) enabled the identification of a wide range of relevant digital environments—including forums, social communities, blogs, chats, and websites—addressing the topic from medical, psychological, experiential, and relational perspectives.

The mapping included 6 online Forums, 15 Facebook groups, 2 Telegram chats, 2 support websites with interactive Q&A sections, 9 websites of active associations and a wide range of professional services related to ART. The selection was carried out with a comparative logic, aiming to cover existing diversity, and took into account thematic centrality, interaction density, and the variety of digital affordances and temporalities offered by each platform (Burgess et al., 2017; Ahlin and Li, 2019). From the exploratory phase onward, particular attention was paid to the forms of interaction enabled by platforms, their technological affordances, modes of communication, degrees of synchronicity, visibility, levels of moderation, access conditions, and the persistence of content. This approach has allowed for an understanding of how reproductive subjectivities and epistemic practices are shaped by the specific characteristics of the platform. The analysis has thus highlighted how digital platforms function as socio-technical devices that not only reflect but also structure the creation of meaning, discursive hierarchies, and visibility in a normatively regulated field such as reproduction.

The selection included spaces with a clear thematic focus on ART, characterized by dense interaction, dialogical and collective communication, deemed relevant, with non-close access, frequent and intense exchanges, and lively interactions.

Conversely, environments inactive or scarcely active, overly generalist, accessible only through advanced profiling or authorization (due to ethical concerns), or oriented toward commercial promotion or professional branding were excluded.

### 3.2 Field access and data collection

Three digital spaces were selected, heterogeneous in structure, communicative configuration, openness, and participation modalities. The first is a thematic ForumFree forum active since 2016, with a public and accessible structure, allowing asynchronous and retrospective observation of discursive transformations. The second consists of two Facebook groups with active moderation and high-frequency interactions.^1^ The third is a Telegram chat characterized by a low barrier to entry and daily exchanges that foster informality and immediacy.

Given the characteristics of the study, the nature of the digital environments, and the sensitivity of the topic, it was decided to adopt a strategy of *passive lurking* through non-participant and covert observation, in line with the principle of minimal intrusiveness and disruption (Shoham, 2004). The observation period covered content published between April and September 2024, ensuring a sufficient temporal depth to capture both recurring dynamics and the diversity of situations experienced by the communities (Reich, 2021; Traianou and Hammersley, 2024).

Access to the three selected online communities^2^ (Kozinets, 2002) was carried out through distinct procedures, aligned with the technical nature and access conditions of each platform.

In public ForumFrees, access was granted without registration; for semi-private Facebook groups, formal access requests were submitted and accepted, while Telegram chats, access was granted according to free entry procedures. The observational stance remained covert throughout.

The corpus of data was constructed through a combination of web scraping and manual archiving, using different methods for each platform, tailored to its specific technical possibilities.

For Telegram, data were extracted using the platform’s native export tool, which enabled the direct downloading of full chat histories. This method provided access to both messages and metadata, ensuring a complete archive of user interactions.

For the analysis of the Facebook group, multiple attempts were made to extract data in an automated manner. Initially, several commonly used tools for web data extraction—such as Instant Data Scraper, Data Miner Premium, Web Data Extractor, a tailored data extraction system prototyped using Python, and an attempt via Facebook’s Graph API—were tested. However, none of them proved effective due to Facebook’s platform-level restrictions on automated data collection.

As a result, data collection from Facebook had to rely on manual archiving through screenshots. While less scalable, this approach ensured the documentation for the qualitative analysis of user interactions, discourse strategies, and narrative practices within the selected group.

To extract data from the selected online ForumFree, a web scraping procedure was developed, as no official APIs were available for content access. While automated tools such as Instant Data Scraper initially proved useful for a preliminary exploration of the site’s architecture, the systematic data collection process was carried out using a dedicated Python script developed specifically for this project.^3^

The script uses the requests library to perform HTTP requests and beautifulsoup4 to parse the HTML pages of the forum. It was designed to recursively navigate the forum’s hierarchical structure—from the homepage through all subsections—identifying and mapping sections, discussion threads, and individual posts. For each message, we extracted the textual content, emojis, quotes, multimedia content, and relevant metadata, including the author, publication date and time, discussion titles, and uniform resource locators (URLs).

The extracted data were stored in a NoSQL document-based database (MongoDB), which allowed for flexible handling of heterogeneous structures and the ability to update or extend data fields without modifying the overall schema. The MongoDB dataset was organized into four collections: authors, sections, discussions, and posts. In the author’s collection, each document is a unique author; in the section’s one, each document corresponds to a discussion; in the discussion’s collection, each document is a single post; and in the post’s collection, each document represents a message. This structure was chosen to facilitate data navigation and to avoid creating a single overly nested collection.

Only posts published during the observation period (April 2024–September 2024) were extracted, ensuring the temporal consistency of the corpus. At the end of the scraping process, a series of data quality checks were performed, based on three main dimensions: accuracy (ensured by the structured and automated nature of the process), completeness (verified through the presence of all required fields and the absence of null values in the database), and consistency (checked by comparing the number of users across databases and verifying the match between quoted messages and recorded user-to-user relationships).

The script was designed to run via the command-line interface (CLI), with the option to set custom start and end date for incremental data collection and periodic corpus updates.

### 3.3 Ethical considerations

In compliance with ethical standards, the research protocol received formal approval from the University of Milano–Bicocca Ethics Committee, in consultation with the institution’s data protection officer.

Given the sensitivity of reproductive health narratives and the intimate nature of the communities under study, a covert, non-participant observational approach was adopted to minimize intrusiveness and avoid interfering with the natural dynamics of digital interaction. Only environments that were publicly accessible or accessible through formal entry procedures (such as approved requests to join Facebook groups) were included. No false intentions were declared, nor were any pieces of information explicitly requested that were omitted, as there were no gatekeeping mechanisms requiring disclosure.

A situated and responsible ethnographic approach was adopted, aimed at minimizing risk and in line with the university’s ethical guidelines. Particular care was devoted to privacy protection: all collected information was thoroughly anonymized (Atenas et al., 2023) and subjected to a fabrication-based rewording process to ensure the non-traceability of the original authors, especially considering the sensitive nature of content related to reproductive health (Markham, 2012).

The researcher’s positionality was continuously reflected upon; with the awareness that even silent observation entails ethical choices. The concealed presence in the field was interpreted in light of the ethics of responsibility (Haraway, 2003; Hesse-Biber, 2013).

### 3.4 Analysis and coding process

The collected corpus of textual data was analyzed qualitatively using NVivo 14 software, following a grounded theory approach (Glaser and Strauss, 1967). The coding process took a circular and recursive form, unfolding across multiple stages of reading, categorization, revision, and comparison, according to an abductive logic. The initial coding framework was developed from a set of exploratory sensitizing concepts and orienting frameworks (Blumer, 1954; Charmaz, 2006), but was progressively reshaped during the analytical work, allowing for the emergence of interpretative categories directly grounded in the data. The interpretative axis was based on a continuous dialogue between empirical content, platform features, and theoretical reflection. The analysis was guided by a detection grid articulated across several dimensions, with specific attention to the impact that affordances exert on the forms of communicative exchange and the construction of collective meanings (see Table 1).

**Table 1:** Analytical dimensions and number of subcategories at the beginning.

Dimension	Subcategories
Dynamics of exchange	8
The medical path of ART	14
The non-medical path of ART	14
The external, economic, and political dimension	5

In particular, the analysis developed around a coding framework articulated along three core thematic dimensions, articulated in subcategories: the medical pathway, the non-medical pathway, and the external and contextual dimension. These areas of inquiry revealed the variety of meanings associated with ART at different stages of the process, conveying the depth and complexity of experiences related to the body, medical treatments, parenthood, and the response to and interaction with social and healthcare contexts.

The first dimension, relating to the medical pathway, focused on narratives of clinical experiences connected to ART, delving into diagnoses, techniques used (e.g., IVF, ICSI, FER, and FO), management of the waiting period, and choices concerning access to treatment and healthcare facilities.

The second thematic dimension concerned the non-medical pathway, exploring experiences outside the clinical setting that are central to reproductive journeys. The focus was directed toward observing key areas as waiting periods, treatment failures, parenthood construction, body and couple representations, donor figures in heterologous ART, sharing or withholding pregnancy news, ambivalences about the chosen path, and challenges in balancing work with medical treatment.

The third dimension examined the external context, including the normative, political, economic, and social environment surrounding the ART process. It focused on sharing the experience within social networks, prejudice and judgment, legitimacy concerns, reflections on ART legislation, access to treatment, and comparisons with alternative paths like surrogacy or adoption.

In addition to these three dimensions, specific and transversal attention was given to the configuration of the dynamics of exchange between users, which are strongly influenced by the technological affordances of the platforms analyzed. The analysis took into account the functional logic of each environment (asynchronous or synchronous, the possibility of direct or nested replies, visibility of participants) and the type of interaction generated (more discursive and community-based, or more reactive or fragmented). Focus was placed on the type of communicative register used (e.g., technical, confessional, ironic, and empathetic), the dynamics of peer exchange, the configuration of key figures in the exchanges (e.g., gatekeepers, mediators, moderators), the variety of user types (lurkers, regular contributors, and opinion leaders), the degree of anonymity or exposure of profiles, the forms of interaction (e.g., reply, mention), the level of detail in personal narratives, and the connections to external networks such as sites, clinics, or professionals.

In fact, to critically interpret the discursive dynamics in digital groups dedicated to ART, the analysis integrated conceptual tools from Actor-Network Theory (Latour, 2005) and Digital Methods (Rogers, 2013). The focus extended beyond decoding user content to include the active role of platforms as technological devices that co-produce the form and meaning of the interaction. In this perspective, affordances are seen as actants, shaping communicative flows, hierarchies, social roles, and self-narration (Caliandro and Gandini, 2019).

Through the combination of grounded theory and affordance analysis, the approach adopted thus held together the levels of the actor and the device, showing how the digital medium is not a passive container but a co-producer of subjectivities and of epistemologies.

The limitations of the netnographic approach must not be underestimated. The partial invisibility of non-textual elements, the non-representativeness of the sample, the fluid nature of digital identities, and the risk of decontextualization require caution. Moreover, the adoption of a non-participant observational approach entails the risk of ethical issues if the analytical process does not ensure procedural rigour, as well as the risk of partial or decontextualized interpretations, thereby limiting the ability to explore subjective meanings and intentions.

Nevertheless, the analysis is based on the assumption that seeking support online is motivated by the absence of a sufficiently welcoming offline environment. Empirically testing this assumption would be valuable to enhance the understanding of users’ needs, the interplay between online and offline forms of support.

## 4 Results

The analysis of empirical materials collected through netnographic observation has allowed for the identification of a plurality of discursive practices, relational forms, and sharing strategies that contextually articulate the experience of the parental quest. The presentation of results unfolds through a qualitative reading of messages, posts and comments, engaging in dialogue with analytical categories prepared prior to data collection and emerging during the exploratory phase of the research.

The netnographic analysis was structured around the four main thematic dimensions, which guided the coding process and organization of empirical materials. As shown in the Table 2, the most represented categories are those related to the non-medical and medical dimensions of ART, followed by interaction dynamics and, to a lesser extent, external, economic, and political factors.

**Table 2:** Distribution of files and references by the netnographic analysis on NVivo software.

Nodes	References
*Exchange dynamics*	*341*
Co-constructed medical competences	31
Connections between the group or forum with clinics or other entities related to the topic	44
The degree of experience sharing	95
Anonymous member	31
Key figures or references in the exchange, gatekeeping or mediation functions	50
Communication register	33
*The medical ART pathway*	*615*
Clinical doubts and information	226
Medicalization (of body and/or identity)	50
Pregnancy detection	9
Medical pathway narratives	38
Hospital or clinic of reference**—**where treatment takes place	129
Post-first positive beta, pregnancy progression	3
Choice process regarding the selected practice	12
Mistrust and obstetric–gynecological violence	40
ART technique	18
Homologous or heterologous	67
Techniques for understanding the clinical situation	23
*The non-medical ART pathway*	*720*
Age	30
Heterologous	41
References and narratives of the relational dimension of the couple	19
Treatment failure	24
Type of support sought in the group (medical, emotional…)	196
Type of support given	39
Waiting for pregnancy post-treatment	52
Reconciliation with work and career	38
Dimension of agency, motivations, resilience	12
Reaction to pregnancy	29
Emotions detected	148
Biographical narratives and self-presentations	57
Narratives on imagined parenthood (fatherhood or motherhood)	23
Religion	12
*External, economic, and political dimension*	*132*
Judgment and prejudice	7
References to the Italian political debate and opinions on it	4
Sharing, if done, with family and friends	32
Accessibility to ART (economic and/or informational)	71

These thematic areas will be discussed in the following sections.

One of the primary transversal findings emerging from the analysis concerns the marked gender dimension permeating all observed platforms. Through indirect indicators—such as users’ names and nicknames, the narrative structure of posts, and explicit references to bodily experiences—a clear predominance of female subjectivities is evident. This finding can be interpreted along multiple lines. On one hand, it reflects a tendency according to which women demonstrate a greater propensity to seek, share, and discuss health-related information online (Stern et al., 2011; Baumann et al., 2017; Nikoloudakis et al., 2018). This predominance may also be attributed to the asymmetric distribution of medical burden in ART: nearly all procedures (monitoring, hormonal stimulation, surgical interventions, cyclical check-ups) are carried out on the female body, even in cases where the diagnosis involves both partners. Indeed, a medicalization of the female body occurs, which, in these cases—with delicate and prolonged procedures—also extends into the realm of identity, as illustrated in various messages.

“It’s a journey where you lose count of the syringes—you’ll get used to it! Good luck!” (Facebook)

“This journey has taken over my life. I plan my job, my holidays, my diet… everything revolves around this” (Telegram)

This asymmetry, expressed at both biological and symbolic levels, is explicitly addressed in many messages. In some instances, it is articulated as a “healthcare burden” falling almost exclusively on the female body, despite the journey often being framed as a shared experience within the couple. Some messages explicitly highlight this disproportion, emphasizing a misalignment between the jointly held project and the greater female exposure to clinical practices.

“I don’t know if it’s the same for you, but in my relationship I’m the one who handles these things because I’m a doctor and he doesn’t have the time.”

“I’m not a doctor, but I still do everything on my own.” (Telegram)

The inequality in physical effort is accompanied by greater exposure to risk, uncertainty, and decision-making burden—factors that help explain the strong female presence within online communities.

The results are structured around five main thematic clusters: (1) the legitimization of the reproductive experience as a narratable experience, (2) the collective construction of situated knowledge between technologies and lived experiences, (3) the discursive performativity of the desire for parenthood, (4) the ritualization of waiting and the uncertainty, and (5) the analysis of how affordances shape user practices. Each section is illustrated with exemplary empirical excerpts and accompanied by a reflection on the dynamics observed.

### 4.1 Legitimization of the experience and normalization of vulnerability

Online communities enable the emergence of meanings and experiences that would otherwise remain inaccessible, as alternative trajectories to naturalistic filiation are still subject to social stigma (Cretella, 2024). Where stigma operates as a socially exclusionary dynamic (Goffman, 1963), individuals are more likely to seek information, recognition, and support in digital environments (Yeshua-Katz, 2018).

Among the main factors driving individuals to engage with these online communities is the need to legitimize their own experience. Narratives are often characterized by an implicit or explicit request for validation of the complexity experienced or reassurance, as seen in the questions posed by many users (e.g., “Why is this happening?,” “Has this happened to anyone else?”), frequently accompanied by intimate and clinically specific details and requests for advice and clinical information.

“Hello everyone. I just did a sperm test and the values are within the normal range except for 98% abnormal forms… Is it a verdict? Please, give me your opinions. I've never seen my partner so down…” (Forum)

In a context marked by constant societal judgment regarding parenthood, cultural taboos surrounding non-“naturalistic” forms of parenthood, and widespread uncertainty surrounding the ART journey. In the absence of a widespread cultural narrative on ART, frequently stigmatized or relegated to the domain of “reproductive failure” online communities function as spaces where the complexity of the journey—marked by uncertainty, desire, fear, hope, pain, and failure—can emerge and be legitimized. Communication within online groups thus becomes a process of normalizing vulnerability, where even the most ambivalent and difficult experiences gain recognition and the possibility of being shared.

An element contributing to the legitimization of experience is the dynamic of peer-to-peer support, which cuts across and is consistently observable within all the platforms analyzed. Responses to users’ questions are often empathetic, oriented toward practical and emotional support: “This happened to me too,” “Hang in there, it will work out.” This type of interaction reinforces a sense of belonging to the community, reduces perceived isolation, and contributes to creating a space accessible for such disclosures.

In fact, isolation and anxiety are among the most frequent experiences reported in the context of ART (Rahimi et al., 2021). Numerous studies confirm that reproductive difficulties are closely associated with high levels of anxiety and psychological stress, both as consequences of infertility and as factors potentially influencing treatment outcomes (Rodríguez-Domínguez et al., 2024).

In these platforms, they emerge as a pervasive and explicit theme.

“So anxious! This morning they’ll call me to let me know if they’ve developed into embryos… Until now I was calm, but now I’m getting nervous… I’m sure you understand me!!” (Facebook)

“I opened up to this friend of mine only because she kept complaining about NON-EXISTENT problems, and at a certain point I couldn’t take it anymore and vented… I prefer to keep quiet and talk about it with you!” (Telegram)

This process involves not only the direct challenges of the reproductive journey—such as clinical uncertainty, medical procedures, or repeated failures—but also the indirect difficulties that accompany it. Users often address structural constraints, including limited economic and informational accessibility, and share concerns about reconciling treatment with work responsibilities, particularly in contexts where their reproductive efforts remain undisclosed in offline environments.

“In my region, there are no facilities affiliated with the NHS for heterologous fertilization, and they don't issue the authorization. Should a family have to take out a mortgage to have a child?” (Facebook)

“I wasn't able to take these sick days abroad in any way. I wrote to INPS but they never responded. No one could give me any information about it, so in the end, I used some leave days” (Forum)

Within this framework, online platforms play a significant role in providing an accessible space where such experiences can be expressed, and partially reworked, allowing individuals to articulate and share a complex experience—often marked by uncertainty, stigma, and silence. The discursive practices emerging in online groups function as tools of collective legitimization and recognition, transforming individual experience into a socially viable trajectory.

### 4.2 Production of situated and peer-based knowledge

Medical information and treatment-related bodily data circulate within digital platforms according to a hybrid logic: on one hand, they refer to a scientific epistemology, employing clinical terminology, therapeutic protocols, references to medications; on the other hand, this information is grounded in experiential knowledge, constructed from individual lived experience, medical advice received during personal care pathways, and an informal repertoire of knowledge transmitted through storytelling, comparison, or hearsay.

In messages, technical language is omnipresent: users speak of “blastocysts on day five,” “transfer,” “progynova,” “beta,” “progesterone,” “hysteroscopy,” “monitoring ultrasound,” “IVF,” or “embryo donation.” This medical jargon, often learned directly during treatment pathways, is transformed into an acquired competence. The lexicon used—rich in technical-medical abbreviations such as “monitorings” or “pick-up”—reflects a process of collective familiarization with medical language, facilitated by its continuous use. These hybrid languages—technical and personal—bridge the epistemic gap between patient and medicine, allowing the expression of vulnerability that might otherwise remain silenced or pathologized offline.

However, such forms of knowledge are not always based on scientifically validated evidence, and clinical information is constantly integrated with bodily sensations, emotions, and subjective experiences. This occurs as users exchange interpretations of clinical results, discuss symptoms and conditions in an effort to identify patterns and possible explanations, and compare different healthcare facilities, thereby contributing to the collective mapping of Italian and international clinics, therapeutic protocols, and medication use practices.

“Beta at 765 on 05/16 and 6789 on 05/22: are these values good?” (Telegram)

“Hysteroscopy, biopsy, and NK plasmacells are always done before a transfer…” “In general, yes, but anyway, plasmacells are usually a response to an infection, and those are treated with antibiotics.” (Facebook)

In this sense, digital platforms do not merely host informational exchanges; they become active spaces of mediation and circulation of knowledge, where the boundary between clinical data and subjective interpretation is constantly ambiguous.

The content produced reflects both continuity with and tension against the offline medical framework, reflecting its shortcomings, communicative asymmetries, and a perceived lack of responsiveness and empathy from healthcare professionals. Notably, a recurring theme is the expression of mistrust—and only to a lesser extent, trust—toward medical professionals, who are at times portrayed as imprecise, unresponsive, or lacking in empathy and attentiveness.

“Hearing more opinions, they all tell you different things. And you get even more confused. Is there anyone who has gone through this same mess?” (Telegram)

“Good evening, does anyone have the name of a good gynecologist in Turin and surrounding areas? Unfortunately, I’ve had bad experiences, so I would like a kind, understanding, and knowledgeable person about assisted reproduction, who is non-judgmental. Thank you.” (Forum)

These digital environments play a crucial role in recirculating and contextualizing medical knowledge, transforming it into a relational resource rather than an epistemological certainty. The knowledge produced is situated and subjectively filtered, representing a form of agency through which patients actively seek to orient themselves and reclaim the meaning of their care journey. A knowledge substrate is generated, not always scientifically validated, but collectively acquired, accessible, and, most importantly, discursively expressible within the community. Medical knowledge is not denied but rather recontextualized and humanized, emerging as a dynamic and cumulative flow (Cossetta and Caliandro, 2013; Fortunati, 2018). However, this reworking often occurs outside of clinical expertise, leading to a partial and sometimes distorted circulation of biomedical information, where scientific data is intertwined with personal beliefs and anecdotal evidence.

### 4.3 Co-construction of parenthood

In online communities dedicated to ART, parenthood is not perceived merely as a biological outcome, but rather emerges as a long, uncertain, and deeply symbolic process that begins prior to a potential pregnancy. The desire for a child appears as a complex identity project, developed over time through cycles of waiting, attempts, disappointments, and renewed hopes. Even those actively involved in the medical process develop a parental identity, interpreting bodily signals and sharing their experiences. Becoming or being a parent thus takes shape as an identity that is already present.

“The wait between the transfer and the test is like being 'half pregnant'. Hope is sky-high, but so is the fear of losing everything.” (Forum)

Three recurring characteristics of parenthood as a process emerge: it is projective, as it is emotionally anticipated before being biologically realized; precarious, as it is traversed by uncertainties, medicalizations, and temporal suspensions; and crystallized, as the desire tends to consolidate into a persistent project over long periods. Periods of waiting become moments of affective imagination construction. The child is symbolically present even before the clinical outcome, and performative language strengthens emotional attachment.

“Every night I imagine a baby in my arms. I know it's not real yet, but I already love them.” (Facebook)

Experiences of ART are deeply embedded in and shaped by the desire for parenthood, which emerges not as a static aspiration but as a temporally and socially constructed process. The desire for parenthood is situated within a biopolitical tension, where two poles coexist—only apparently opposed: on one hand, the active pursuit of a child through medical assistance as an act of self-determination; on the other, the crystallization of the desire for parenthood as an affirmation of a cultural norm (Cavarero and Restaino, 2002). The medicalization of bodies and the duration of the process contribute to making the desire for parenthood omnipresent, daily, and temporally extended.

Regarding this point, we observe that the biological connection remains a strong symbolic reference even when alternative pathways to naturalistic filiation are undertaken. This is particularly evident in narratives concerning donor gametes or embryo donation, which reveal an ambivalent dimension: while some open the possibility of redefining parenthood in terms of intention and care; others testify to the emotional difficulty of relinquishing genetic transmission.

“Now they’re suggesting egg donation or embryo adoption. But emotionally, it’s extremely hard to accept. How can one give up their own genetic heritage?” (Forum)

These narratives not only share personal experiences but also reflect and actively contribute to the cultural and discursive construction of parenthood. On this topic, an important function performed by online communities is the redefinition of failure: it is not stigma, but a shared and reworkable experience. Personal accounts function as resources for those undergoing the same journey:

“I don’t want to mislead you, but it happened to me too, and my beta levels turned out positive… the uterus expands, so the embryo has difficulty implanting deeply” (Facebook).

Furthermore, parenthood is performed even by those who have “made it,” who often remain active in the group to “give hope” to those still waiting, thus constructing an archive of experiences that becomes a collective resource. Pain, failure, and uncertainty are not denied, but transformed into opportunities for support.

“One year ago I was exactly in your place… today my baby girl is sleeping next to me. Don’t give up” (Forum).

Online communities represent discursive spaces where parenthood is conceived as a reality in the making—imagined, desired, and narrated before it is realized. Through a plurality of situations and trajectories, these platforms render fragility speakable and legitimize the conception of the parental project, even when temporally and spatially dislocated from its factual realization.

### 4.4 The ritualization of waiting

Waiting is one of the most salient and recurrent dimensions in the narratives of users participating in online communities dedicated to ART. The anticipation of responses, beta-human chorionic gonadotropin (beta-hCG) results, ultrasound scans, the menstrual cycle, the next stimulation cycle, or embryo transfer becomes a suspended time—emotionally dense and marked by ambivalent feelings—which participants attempt to navigate and manage through ritualized narrative practices, also as a means of resisting the isolation often inherent in these journeys.

The temporal gap between the desire for parenthood and its realization is managed through segmentation of the ART journey into distinct phases, providing guidance and a partial sense of control. Users structure their experience around specific moments, such as medical visits or embryo transfers, using shared temporal markers (e.g., “+5 after the transfer”), which intertwine with the monitoring of bodily signals, interpreted both individually and collectively.

Online communities become crucial resources during waiting periods, through practices such as sharing posts on key days, seeking clinical information and feedback on physical sensations (“Is it normal to have these spots?”), discussing therapeutic choices, sharing results (“Beta 14, I still believe”), and exchanging messages of encouragement. These digital spaces counteract suspension, offering forms of emotional support and mutual understanding, while creating cultural tools to manage uncertainty, collectively processing the anxiety and worry that permeate ART pathways (Yeshua-Katz, 2018).

This shared temporality constitutes one of the foundational elements that bind and structure online communities, where individual trajectories intersect in common calendars. In contrast to the objective parameters and the often opaque and uncertain timelines of reproductive medicine—which tends to adopt a performative logic, reducing bodies to binary criteria of success/failure (e.g., laboratory results, test outcomes)—the ritualization of waiting within online communities provides individuals with a space to confront and make sense of uncertainty. This process contributes to transforming suspended time into a shared and narrativized temporality.

A significant aspect of these waiting phases is the redefinition of the body, understood in terms of biological efficiency and continuously observed, questioned, and narrated as an object of constant monitoring. Patients refer to specific bodily sites, fragmenting them—the uterus, breasts, pelvic pain—and describe them in relation to the potential meanings associated with their clinical phase.

“My breasts are sore—could this be a good sign?” (Facebook)

“I have cramps in my lower abdomen, like before my period, but this time I hope it’s different.” (Forum)

The distinctive temporality of ART pathways contributes to consolidating the pervasive process of bodily medicalization, already mentioned, which tends to become identitarian. The body is not only medicalized in its physiological processes, but also in women’s subjective self-perception: individuals come to define themselves through clinical markers (“low AMH,” “retroverted uterus,” “hyperstimulation”), internalize diagnostic logics (“I’m a difficult patient,” “I do not respond well to stimulation”), and intertwine identity with procedural milestones (“I’m in downregulation,” “Today is transfer day”). Expressions such as “I hope it implants this time” or “I had the egg retrieval but only two mature oocytes” illustrate how technical language becomes incorporated into personal narratives of self and body. Notably, self-presentations often reflect this integration:

“Good morning, I’m a 37-year-old woman. On October 14, I had the transfer of a day-5 embryo. On October 23, I took my first beta test, it was positive. Shortly after, I started having some spotting and in the seventh week, on November 15, I lost the pregnancy.” (Forum)

This internalization of medical codes, intensified during the waiting phases, has ambivalent effects. On one hand, it fosters awareness and agency, allowing patients to navigate the clinical pathway; on the other hand, it can generate feelings of guilt, inadequacy, and alienation, especially in the face of failures. Identity is thus built in a delicate balance between agency and vulnerability, knowledge and dependence, empowerment and frustration (Cossetta and Caliandro, 2013). Waiting periods in ART are not empty times, but significant spaces, supported by discursive practices, digital rituals, and community coping strategies, which contribute to the creation of an alternative language and temporality, providing space for a process that is often invisible.

### 4.5 Platform affordances and configurations of interaction

The netnographic analysis shows how the three digital platforms are not neutral environments but rather devices that deeply shape the discursive and relational practices of users. The specific technical architectures, algorithmic logics, and modes of participation of each platform determine specific communicative vocations, influencing self-narrative styles, the temporality of the shared experience, agency dynamics, and mechanisms of interactional regulation (Caliandro and Gandini, 2019). More specifically, the three platforms enable distinct modes of self-presentation. On ForumFree, when introducing themselves, users often adopt long biographical posts that reconstruct their clinical history, age, relationship status, and previous attempts, turning their trajectories into consultable “public diaries.” In Facebook groups, self-descriptions are usually shorter and often event-based, and are shaped by the semi-public nature of the setting and by the visibility of personal profiles. In Telegram chats, by contrast, self-presentation is fragmented and highly situated in the flow of conversation: users refer to themselves mainly through micro-events of everyday life, producing an ongoing, collective stream of embodied self-talk. These platform-specific communicative regimes thus contribute to configuring different possibilities for narrating the self and for negotiating what it means to be “a patient” in ART.

ForumFree, Facebook, and Telegram act as *actants* that contribute to the construction of a digital ecosystem around ART, facilitating different configurations of subjectivity, relational dynamics, and knowledge production, and promoting a collective process of legitimation and cultural production of the reproductive experience (see Table 3).

**Table 3:** Comparative analysis of platform affordances.

Platform	Communicative vocation	Temporality	Moderation	Narrative form	User agency
ForumFree	Experiential archive	Horizontal^a^, reflective	Self-managed	Long-form, structured, retrievable	Narrative, informative
Facebook	Protected lounge for emotional exchange	Semi-synchronous, event-based	Admin-moderated	Episodic, affective, symbolic	Emotional, relational, performative
Telegram	Daily diary against loneliness	Vertical^b^, real-time	Self-moderated, implicit	Dialogic, instantaneous	Empathic, ritual, co-regulative

a By “horizontal time,” we refer to the ability to navigate through posted events regardless of when they were published, thanks to the archive-based structure of the forum, which allows for a non-linear view of exchanges.

b “Vertical time” refers to a platform architecture that does not fix messages but instead generates an ongoing exchange, as seen in Telegram chats, where conversations flow in real-time without a static chronological order.

This overview shows how each platform facilitates different configurations of subjectivity, relational dynamics, and knowledge production, contributing to the construction of a digital ecosystem around ART. Within this ecosystem, experiences give shape to a collective process of legitimization, sharing, and cultural production of reproductive experience.

The comparative reading of ForumFree, Facebook, and Telegram highlights how each platform enables specific communicative regimes and participatory models.

#### 4.5.1 ForumFree: historical and consultable archive

On the ForumFree-hosted forum, key affordances include a clear organization of threads, the persistence of content, and the ability to navigate through thematically organized topics. This configuration promotes extended, reflective, and structured forms of narration. Users frequently produce detailed accounts of their ART journeys, often developed as “public diaries” that remain accessible and consultable over time. Comments follow a sequential logic and, even in highly populated threads, a degree of narrative coherence is maintained. Anonymity, ensured by the use of nicknames, encourages the emergence of intimate content—sometimes with a therapeutic intent.

The forum takes on the characteristics of a reflective and structural archive, marked by a horizontal and cumulative temporality. ART trajectories have been accumulating over the years, generating a shared knowledge base that is both technically informed and affectively codified. While rooted in subjective experience, the narratives acquire epistemic value for the community, contributing to the circulation of information on medical protocols, therapeutic practices, healthcare facilities, and psychological dynamics associated with ART.

The archival function of the forum preserves a collective memory that strengthens the continuity of the community discourse, acting as a device for discursive sedimentation. In addition to archiving content, the forum facilitates distributed knowledge, integrating medical, pragmatic, and social dimensions, thus becoming a space for self-education and mutual orientation.

#### 4.5.2 Facebook: virtual waiting room

The Facebook groups observed can be understood as virtual medical waiting rooms—semi-public spaces regulated both by the presence of moderators and by the algorithmic architecture of the platform. The environment supports semi-synchronous emotional interaction: posts are generally brief and refer to immediate events (“Today I had my egg retrieval,” “I’m extremely anxious about tomorrow’s beta test”), often receiving comments within a few hours, though they may also be revisited after several months. However, they neither follow the continuous and unstructured flow typical of Telegram chats nor the articulated and orderly structure of ForumFree, which facilitates easier navigation.

Facebook features affordances oriented toward visibility and reactivity. The platform’s algorithmic logic privileges posts with high engagement, comments, and reaction functions (likes, hearts, emojis) to incentivize immediate exchanges. Within the groups, personal identity can be revealed, so the option to post as an “anonymous member” is sometimes used, enabling users to engage while maintaining their privacy. Shared rhetorical formulas, emotional abbreviations, emojis, and images contribute to the construction of a codified language, reflecting a collective and consolidated competence—both medical (e.g., “crossed betas”) and emotional (e.g., “In becco alla ciko!^4^”)—which strengthens the sense of belonging to an otherwise invisible community.

The platform’s structure and specific affordances position it somewhere between Telegram and ForumFree, both in terms of interaction timing and the kind of support sought. Awareness of a potentially broad audience diminishes the sense of intimacy compared to Telegram, while the—albeit somewhat cumbersome—possibility of revisiting past discussions makes it more similar to the ForumFree model.

#### 4.5.3 Telegram: daily diary against loneliness

Telegram embodies a distinctive communicative logic, closer to the synchronous and informal nature of everyday chats. The affordances promote fast, non-hierarchical, and continuous interaction and messages are short, numerous, and written in real time, following a vertical logic, that fosters strong emotional engagement. The platform’s interface does not facilitate chronological reconstruction of conversations. The absence of thematic threads and the cascading, unstructured nature of exchanges encourage the immediate expression and the emergence of spontaneous peer-to-peer support practices.

The observed chat functions as a daily diary against loneliness and in this context, forms of situated micro-narration emerge, where personal experience is intertwined with practical exchanges (“Which gynecologist would you recommend in Rome?”), emotional outbursts (“I cannot take it anymore”), and urgent requests for reassurance (“I have cramps, is that normal three days after the transfer?”). The topics discussed confirm a collective narration of the everyday (Beer and Burrows, 2010): alongside the medical journey, conversations also touch on films, daily activities, and small repeated gestures while waiting.

Telegram thus functions as a space characterized by high levels of situated empathy, where mutual support is fostered through frequent interactions. Responsibility is collectively distributed and expressed through tone modulation—gentle and calm registers—and through the shared construction of an inclusive, non-judgmental, and self-moderated environment. This type of flux can lead to information overload or the marginalization of certain topics, which may become obscured within the continuous flow of conversation.

These three configurations reveal that digital affordances are not merely technical backdrops, but active devices of mediatization that shape both the conditions under which communities form, interact, and produce meaning, and the ways in which reproductive experiences are narrated, understood, and negotiated. This diversity underscores the importance of a multi-sited and media-sensitive approach to the study of digital communities engaged with ART, capable of capturing the complexity of how meanings, identities, and knowledges are produced and circulated across platforms (Yeshua-Katz and Hård af Segerstad, 2020).

## 5 Discussion and conclusion

ART experiences are multimodal phenomena, where the clinical component, taking place offline, is deeply intertwined with symbolic, affective, ethical, and relational dimensions that unfold also in online spaces. In Italy, the lived experience of infertility and medicalized parenthood—specifically motherhood—occurs within a strongly normative social and cultural framework, marked by taboos, gender expectations, and latent forms of stigma, which contribute to keeping these experiences partially invisible and often push them into digital spaces (De Sanctis et al., 2020).

Within this context, the digital space plays a fundamental role in compensation, expression, and collective reflection. Digital environments are initially experienced as liminal spaces, capable of welcoming, reworking, and amplifying experiences often left unspoken in the public discourse. Yet, for many users, these spaces soon become part of everyday life. Telegram, in particular, functions as a continuous, daily environment, whereas Facebook and ForumFree retain a more intermittent and liminal dimension. A totalizing process such as ART requires spaces that are always available to land on. This is what online communities become: a space outside of space, for a temporality outside of time (Segatto and Dal Ben, 2017).

In the absence of offline spaces for dialogue and self-expression specifically designed to address infertility collectively and safely, many users turn to digital environments in search of support. While these settings can activate processes of horizontal knowledge-sharing and forms of individual and collective agency, it is necessary to critically examine issues of access and the broader implications for social justice. The lack of medical information and psychological support in offline contexts drives many people toward digital platforms; however, significant barriers persist there as well—cultural, linguistic, and related to the digital divide. These obstacles limit participation and render access to ART uneven, particularly affecting groups already marginalized by legal or healthcare systems—such as lesbian, gay, bisexual, transgender, or queer + (LGBTQ+) individuals, people living in poverty, migrants, and non-Italian speakers. In this scenario, infertility emerges not only as a biomedical condition but also as a factor of social exclusion, embedded in dynamics that may take on indirectly eugenic traits (Cretella, 2024). Moreover, even for those who can easily access online spaces, it is important not to overlook the fact that the support and information available are not of a medical or psychological nature. This raises the risk of misinformation and the reinforcement of stereotypical narratives. While digital communities often provide crucial emotional validation and shared experiential knowledge, they are not substitutes for professional care. In these contexts, advice is largely anecdotal, unverified, and shaped by the dominant voices within the group. As such, online interactions may unintentionally perpetuate normative expectations around gender, success, and perseverance, or circulate inaccurate understandings of medical procedures and outcomes.

ART is thus revealed as an ontologically complex and technologically mediated experience. The clinical journey—from hormonal stimulation to embryo transfer—is deeply intertwined with digital practices of narration, self-monitoring, and mutual recognition. As Floridi (2014) argues, the onlife condition dissolves the boundaries between the online and offline, making digital infrastructures intrinsic to contemporary subjectivities. Lupton (2018) further emphasizes the embodied and affective nature of digital interactions, whereby self-tracking, emotional expression, and support-seeking become core dimensions of health and care.

The three platforms analyzed—ForumFree, Facebook, and Telegram—highlight how different affordances shape distinct discursive and relational regimes (Rogers, 2013). ForumFree offers a consultable archive structured by thematic threads; Facebook allows episodic, emotion-centered exchange within a semi-private environment; Telegram fosters real-time, intimate, and unmoderated communication. These media act as socio-technical agents (Latour, 2005), co-constructing meanings and practices around ART.

Such affordances are not neutral containers: they actively shape what can be said, how it is shared, and by whom. The persistence of ForumFree’s threads, for example, enables detailed cycle diaries and cumulative memory work, whereas the speed of Telegram exchanges sustains ongoing peer coaching and affective synchronization. Facebook’s semipublic nature supports the visibility of success stories while also triggering dynamics of comparison and emotional regulation. As Cossetta and Caliandro (2013) remind us, platform architectures contribute to defining the symbolic thresholds of inclusion and exclusion in digital publics.

Users in these communities integrate clinical knowledge with embodied experience, generating what Clarke et al. (2003) term “vernacular biologies”: lay epistemologies grounded in lived reality. Posts often combine medical terminology with subjective interpretation: “My progesterone was low but I still felt implantation cramps,” or “My beta is 230 on day 12—is that normal?.” This blending of registers, documented also by Cossetta and Caliandro (2013), reveals a distributed epistemology where expertise is collectively constructed.

A central axis of this co-construction is the ritualization of waiting. In ART, time is fragmented into phases—stimulations, transfers, betas—each loaded with symbolic and emotional weight. These phases become occasions for collective participation: countdowns, symptom tracking, peer reassurance. As Bach (2022) argues, these affective temporalities fold embodied experience into practices of hope and fear. The temporal suspension between intervention and outcome is filled with narrative density and relational scaffolding.

Moreover, waiting becomes a shared temporality—a temporality of resonance, where the repetition of similar emotions across bodies and contexts produces a form of relational knowledge. The expression of symptoms, doubts, and expectations during these liminal phases generates what Segatto et al. (2023) describe as a synchronization of affective rhythms. In this way, the personal is collectivized, and the temporal experience of uncertainty becomes a central form of community-making.

Parental desire, in this digital ecology, is not presupposed but performed. Rather than an innate drive, it emerges as a relational project, continually reaffirmed through discourse and interaction. Users frame themselves as mothers-in-waiting, patients-in-transition, subjects of perseverance. As Ahmed (2004) notes, emotions circulate, accumulate, and produce alignments. Within these groups, such affective economies structure legitimacy: to persist, to feel, to narrate is to belong. Lee (2017) observed how perseverance becomes a moral economy, valorizing those who remain in the journey despite adversity.

This desire is constantly shaped, expanded, and re-inscribed by collective narratives. The digital storytelling practices reinforce shared imaginaries of success, resilience, and worthiness, often articulated through metaphors of battle, pilgrimage, or destiny. The narrative grammar of ART includes arcs of hope, setbacks, and symbolic rebirths. These narrative patterns provide users with scripts for meaning-making, while also constructing normative models of the “good patient,” the “deserving mother,” or the “strong woman.” This discursive infrastructure, while supportive, may also exert subtle pressures and exclusions, particularly for those whose journeys diverge from the dominant script.

This study also foregrounds the performative dimension of digital participation. Through nicknames, avatars, narrative tropes, and discursive conventions, users build recognizable identities within the group. Hashtags, emojis, and ritualized expressions function as what Caliandro and Gandini (2019) calls “invisible hashtags”: markers of alignment and affective resonance. These micro-practices foster group cohesion and reinforce norms, but also produce exclusions: those who deviate from dominant scripts—by discontinuing treatment, for example—often disappear from the narrative field.

Digital platforms do not merely reflect ART experiences; they help constitute them. As Lupton (2018) and Selwyn (2019) emphasize, the digital is not external to social life but embedded in its very formation. The interactions observed here suggest that ART is lived as much through screens and chats as through needles and clinics. ForumFree becomes a library of collective endurance; Facebook, a waiting room of mutual recognition; Telegram, a virtual home for daily vulnerability.

From this emerges a broader redefinition of kinship and legitimacy. Narratives of donor gametes, single motherhood, or embryo adoption circulate alongside traditional narratives of biogenetic reproduction, often revealing ambivalence and tension. The cultural model of the genetic child remains dominant, yet alternative configurations are actively negotiated. As Thompson (2005) and Mamo (2007) note, ART enables new ontological choreographies of parenthood, where intention, affect, and community may override biology.

This research thus makes four key contributions. First, it shows that ART is not only a biomedical process but a digitally mediated, emotionally saturated, and socially distributed experience. Second, it demonstrates how parental desire is shaped through collective narration and performance, rather than existing as a pre-social given. Third, it highlights the role of platforms as active agents in structuring subjectivity, legitimacy, and knowledge. Fourth, it reveals how storytelling functions as a mode of symbolic survival, enabling users to transform suffering and uncertainty into continuity, recognition, and hope.

Methodologically, the study underscores the potential of netnography for analyzing intimate, medicalized, and technologically embedded experiences. Following Kozinets (2017) and Hallett et al. (2014), the approach captures both discursive content and relational dynamics, while remaining attentive to ethics and positionality. By “following the medium” (Rogers, 2013) and “following the natives” (Latour, 2005), the research illuminates the intersection of media infrastructures and user practices.

Moreover, netnography allows for the observation of emergent socialities and vernacular expressions of medicalized identities. In tracing how users reframe medical categories (e.g., “low responder,” “chemical pregnancy”) through affective and narrative lenses, the method captures the semiotic and symbolic labor of users. The study demonstrates that digital discourse is not merely reflective, but constitutive of how users feel, know, and act in relation to their reproductive path.

Nevertheless, the research is not without limitations. The reliance on non-participant observation, while ethically motivated, has limited access to the deeper layers of meaning that might emerge in direct interaction or interview-based engagement. This choice, while ensuring minimal intrusion, has precluded the possibility of capturing tacit knowledge, ambivalences, or contradictions that participants might not express explicitly in posts. In addition, the platform-specific nature of the data collection has excluded other emerging spaces (such as Instagram or TikTok), which may host younger or differently situated narratives. The textual focus of the analysis, albeit rich, leaves out multimodal dimensions of digital expression—such as the role of images, videos, voice messages, and memes—which play an increasingly central role in online self-representation and community building.

Furthermore, the communities observed reflect the experiences of digitally literate, self-selected individuals, primarily cisgender women who actively seek peer support. This introduces a bias that does not account for ART experiences of other marginalized or less visible groups, including male partners, LGBTQ+ individuals, people with disabilities, and those with limited access to digital resources. The study also does not fully explore how language, regional disparities, and legal status shape participation and visibility within digital spaces, potentially reinforcing pre-existing inequalities.

These limitations point toward fertile directions for future research. First, greater attention should be devoted to the visual and esthetic dimensions of ART-related storytelling. How do images of medications, ultrasounds, embryos, or symbolic artifacts (candles, amulets, bracelets) participate in the performativity of desire and community belonging? Second, there is room for comparative analysis across national and cultural contexts. How do discourses and practices around ART vary in countries with different legal frameworks, religious traditions, and levels of digital infrastructure? Third, future studies could explore participatory methodologies, such as co-design, digital ethnography with user feedback, or collaborative storytelling projects that amplify underrepresented voices.

An intersectional lens (Crenshaw, 1991) is essential in these developments. Socioeconomic status, ethnicity, age, migration background, and sexual orientation all influence not only access to ART but also the conditions of online participation, legitimacy, and representation. Investigating how these axes of difference intersect within digital communities can shed light on hidden hierarchies and subtle exclusions. Finally, further research should investigate the interplay between digital and offline support systems. How do online experiences complement or contrast with interactions in clinics, therapy rooms, or family networks? What happens when users exit digital communities—whether due to pregnancy, fatigue, or disenchantment? Mapping these transitions may help understand the emotional ecologies of ART beyond platform boundaries.

In conclusion, ART is not only a clinical endeavor but a relational, symbolic, and mediated one. The experiences explored in this study reflect the entanglement of body, desire, technology, and narrative in contemporary parenthood. Digital platforms, far from being neutral spaces, act as co-producers of reproductive subjectivities. Through shared stories, collective rituals, and vernacular epistemologies, users make sense of uncertainty and reclaim agency in a process often marked by silence and exclusion. This points to a broader transformation in the ways reproductive lives are imagined, shared, and made meaningful through digital participation and collective narration.

## Data Availability

The datasets for this article are not publicly available due to concerns regarding participants anonymity. Requests to access the datasets should be directed to the corresponding author.

## Footnotes

1 The key difference between the two Facebook groups lies in their moderation styles, which shape the groups’ dynamics. In one group, moderation is more hierarchical, with a figure potentially linked to ART services, fostering a more structured environment. In the other group, moderation is limited, focusing on tone and discourse management, promoting a more horizontal, peer-driven atmosphere with higher participation and more exchanges.

2 *Online communities* extend traditional community (Muniz and O’Guinn, 2001) dynamics through digital connections, enabling individuals with common goals to interact. Key criteria include interactivity, multiple communicators, a shared space, and continuity (Jones, 1997). These communities persist as long as users feel a sense of belonging and peer support (Schau et al., 2009).

3 Special thanks to Giulia Pasqualini and Lorenzo Cantarutti, Data Science Master’s students at the University of Milano-Bicocca, for designing and developing the custom ForumFree data extraction system.

4 The abbreviation of “cicogna,” which means “stork,” symbol of fertility.
